# Determinants of Mortality among Adult HIV-Infected Patients on Antiretroviral Therapy in a Rural Hospital in Southeastern Nigeria: A 5-Year Cohort Study

**DOI:** 10.1155/2014/867827

**Published:** 2014-08-06

**Authors:** Kelechi N. Eguzo, Adegboyega K. Lawal, Cynthia E. Eseigbe, Chisara C. Umezurike

**Affiliations:** ^1^School of Public Health, University of Saskatchewan, Saskatoon, SK, Canada S7N 5E5; ^2^Department of Obstetrics and Gynecology, Nigerian Christian Hospital, Aba, Abia State 450001, Nigeria; ^3^Department of Laboratory Services, Nigerian Christian Hospital, Aba, Abia State 450001, Nigeria

## Abstract

*Background*. Study examined the determinants of mortality among adult HIV patients in a rural, tertiary hospital in southeastern Nigeria, comparing mortality among various ART regimens. *Methods*. Retrospective cohort study of 1069 patients on ART between August 2008 and October 2013. Baseline CD4 counts, age, gender, and ART regimen were considered in this study. Kaplan-Meier method was used to estimate survival and Cox proportional hazards models to identify multivariate predictors of mortality. Median follow-up period was 24 months (IQR 6–45). *Results*. 78 (7.3%) patients died with 15.6% lost to followup. Significant independent predictors of mortality include age (>45), sex (male > female), baseline CD4 stage (<200), and ART combination. Adjusted mortality hazard was 3 times higher among patients with CD4 count <200 cells/*μ*L than those with counts >500 (95% CI 1.69–13.59). Patients on Truvada-based first-line regimens were 88% more likely to die than those on Combivir-based first line (95% CI 1.05–3.36), especially those with CD4 count <200 cells/*μ*L. *Conclusion*. Study showed lower mortality than most studies in Nigeria and Africa, with mortality higher among males and patients with CD4 count <200. Further studies are recommended to further compare treatment outcomes between Combivir- and Truvada-based regimens in resource-limited settings using clinical indicators.

## 1. Introduction

In 2008, it was estimated that 33.4 million people were living with HIV/AIDS worldwide, with about 70% of those in sub-Saharan Africa [1]. Poor access to treatment services, poverty, and ignorance have contributed to the high mortality associated with this disease in the region [2]. Following the discovery of the disease in Nigeria in 1986, there has been significant development in efforts to combat HIV/AIDS, including scaling up of antiretroviral treatment (ARTs). This increased the number of people accessing ARTs from 10,000 (2002) to 300,000 (2010) [3]. Nigerian Christian Hospital is one such treatment facility that was activated in August 2008 in southeastern Nigeria.

However, few studies have been conducted to investigate the determinants of mortality for people using ART services in southeastern Nigeria. Onoka et al. studied HIV programs in this region and reported loss-to-follow-up (LTFU) rate between 11 and 32.8% [4]. They also showed that mortality among patients in the programs reviewed ranged from 34.2% to 56.7%. Males and patients CD4 cell count ≤200 had higher rates of LTFU; this study did not explore the pattern of mortality based on drug treatment. Ogoina et al. studied the pattern of morbidity and mortality among hospitalized patients on ARTs [5]. This study estimated a mortality rate of 31.9%, mostly due to disseminated tuberculosis and sepsis. Death was higher among males and people with lower CD4-cell count. There was no statistical difference in mortality based on participation in ART program. Some researchers (Bedavid et al. and Valen et al.) have also suggested that Truvada-based 1st line regimen had better survival profile than Combivir-based 1st line drugs [6, 7]. But, there is inconclusive evidence about the superiority of Truvada-based regimens over Combivir-based regimens. Truvada-based regimens have been found to be associated with less toxicity and better dosing and enhance overall CD4 cells recovery [6, 7].

Given the significant investment in various treatment regimens in our center, this study sought to research into the determinants of mortality for people receiving ARTs. A better knowledge of the prognostic factors will allow tailored follow-up and better-targeted interventions in high-risk patients, thus improving survival. The objective of this present study was to analyze survival and to identify determinants of mortality in HIV-infected adult patients taking ARTs in a rural Nigerian hospital in the last 5 years. This study aimed to examine the relative survival of patients on Combivir- and Truvada-based regimens.

## 2. Methods

### 2.1. Study Setting and Participants

This study was conducted in Nigerian Christian Hospital (NCH), a 110-bed faith-based hospital affiliated with the Church of Christ and International Health Care Foundation. Based in a rural village, the hospital is a major health provider to people from across southeast and south-south Nigeria. It offers specialist services in Surgery, ophthalmology, oncology, and internal medicine as well as obstetrics and gynecology.

NCH commenced antiretroviral treatment (ART) program in August 2008 under AIDSRelief consortium led by Catholic Relief Services. Presently it has enrolled more than 1,900 people living with HIV/AIDS in its program. Services in this program include provision of voluntary counseling and testing, prevention-of-mother-to-child transmission (PMTCT), pediatric and nonpediatric ARTs, as well as immunology. Most of the patients enrolled in the program were recruited through antenatal care, voluntary testing, referral, and in-patient hospitalization. A team of nurses and community health officers as well as monitoring and evaluation workers ensured patient followup and tracking of appointments.

All patients were considered eligible for ART if they were in WHO clinical stage 3 or 4 irrespective of CD4 cell count; if they were pregnant irrespective of CD4 count; and if they had a CD4+ cell count ≤350 cells/*μ*L. Other details of entry criteria were per the National and WHO guidelines [3, 8]. Patients were given appointments ranging from monthly to quarterly, and were monitored for adherence through home visits and frequent phone calls as well as attendance in support group meetings. All patients were assigned unique identification numbers and were monitored through IQ Care software.

This is a retrospective, cohort study of all patients aged 15 years or older who are on ART (1st and 2nd line) in Nigerian Christian Hospital between August 1, 2008, and October 25, 2013. The inclusion criteria were as follows: (1) subjects must be HIV positive, ≥15 years of age, and enrolled at NCH, and (2) subjects have commenced ART. It excluded patients who were transferred to other treatment facilities during the period. Approval for the study was obtained from the Board of Nigerian Christian Hospital. Patient confidentiality was maintained by using anonymized data extracted from the database. Variables considered in our analysis were the patient's biological sex, age, baseline CD4 count, and current treatment regimen.

### 2.2. Treatment, Monitoring, and Endpoints

Various highly active antiretroviral therapy (HAART) was used in this study period. First line treatment comprised combination of lamivudine-3TC and zidovudine-AZT as* Combivir*, with either nevirapine (NVP) or efavirenz (EFV). Another first line regimen was* Truvada* (emtricitabine/tenofovir-FTC/TDF) with either nevirapine (NVP) or efavirenz (EFV). Regimen choice was subject to availability, baseline CD4 count, pregnancy status, TB coinfection, and previous ARTs exposure. Patients with CD4 ≤ 400 cells/*μ*L were given Cotrimoxazole prophylaxis 960 mg daily. After the initial 2 weeks of daily drug administration, antiretroviral drugs were dispensed on a monthly basis. With consistent adherence and followup, patients who were considered stable were given 2-monthly appointment schedules. Second line therapy was given to clients who had clinical or virologic failure on first line, comprising a combination of Lopinavir/ritonavir (Kaletra, Aluvia-ALV) with either Truvada or Combivir. These treatments were categorized as Combivir-based 1st line (3TC/AZT with EFV or NVP); Truvada-based 1st line (TDF/FTC with EFV or NVP); Combivir-based 2nd line (3TC/AZT with ALV), and Truvada-based 2nd line (TDF/FTC with ALV).

For the purpose of this study immunologic classification of the disease was done using CD4 count, based on WHO criteria (see Table 1) [9]. Patients with CD4 ≥ 500 were classified as “not-significant”; 350–499 as “mild”; 200–349 as “advanced”; and <200 as “severe.” Further classification was based on age as 15–24 years; 35–44 years, and ≥45 years. This is also in line with classification used in peer-reviewed literature [2, 6, 10].

The endpoint in our study was mortality from all causes, although the study did not consider individual causes of death. Deaths were registered from hospital records or reported by relatives through home visits or phone calls. Where the actual date was unknown, the date of last follow-up visit was used. Right censored events were subjects lost to followup (LTFU), defined as a patient who missed appointment for more than 6 months and could not be traced. Their date of last follow-up visit was used as the censoring date. Also, patients who were alive at the end of the study period were censored on October 25, 2013. Patients who were transferred to other facilities during this period were excluded in the analysis.

### 2.3. Statistical Analysis

Data analysis was done using SAS version 9.3. (SAS Institute Inc., Cary, NC, USA). Kaplan-Meier models were used to estimate survival during ART care and log rank tests to compare survival curves among different groups. Cox proportional hazards models were used to identify independent predictors of mortality and calculate hazard ratios. The survival time was calculated in months using the time interval between the date of commencing ART and (1) the date of event (death) or (2) date of censoring. Univariable Cox regression analysis was performed for the following baseline variables: sex, age group, baseline WHO CD4 category, and drug combination. Baseline variables significant at *α* < 0.2 level in univariable analysis were included in the final multivariable model (see Table 2). All other analyses were two-sided and level of significance was set at *P* < 0.05.

## 3. Results

A total of 1069 patients were included for this study, comprising 738 females (69%) and 331 males (31%). The median age in the study was 37 years; males 42 years and females 35 years, respectively. Median survival time could not be estimated as over 90% of the patients were censored. A total of 78 (7.3%) patients were reported dead in the 5-year period after enrollment in the program, while 991 (92.7%) were censored. The dead comprised 42 females (54%) and 36 males (46%). Based on age, the dead comprised 79.5% of persons aged above 34 years; 15–24 years (1.3%); and 25–34 years (19.2%). The reasons for censorship were that 168 (15.6%) were lost to followup (LTFU) and 820 (76.9%) were alive, at the end of the 60-month follow-up period. The LTFU consisted of 58 males (34.5%) and 110 females (65.5%). Loss-to-followup was the most common among patients aged 25–34 (36.9%) and 35–44 years (36.9%) and the lowest among those aged 15–24 years (4.8%). At the end of the study period, 820 patients were still on follow-up, comprising 561 females (70.8%) and 231 males (29.2%). Table 2 summarizes the characteristics of patients in this study.

**Figure 1:**
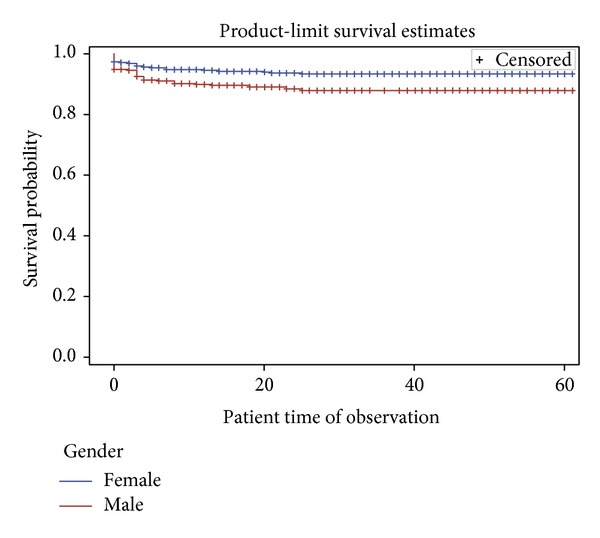
Survival curves based on gender among patients in NCH.

**Figure 2:**
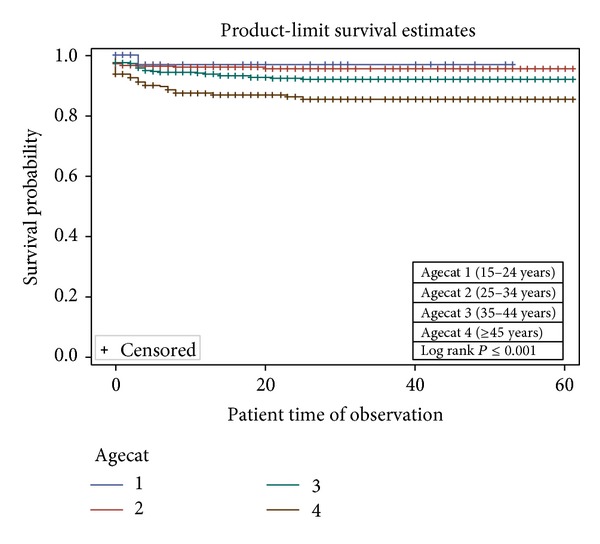
Survival curves based on age group among patients in NCH.

**Figure 3:**
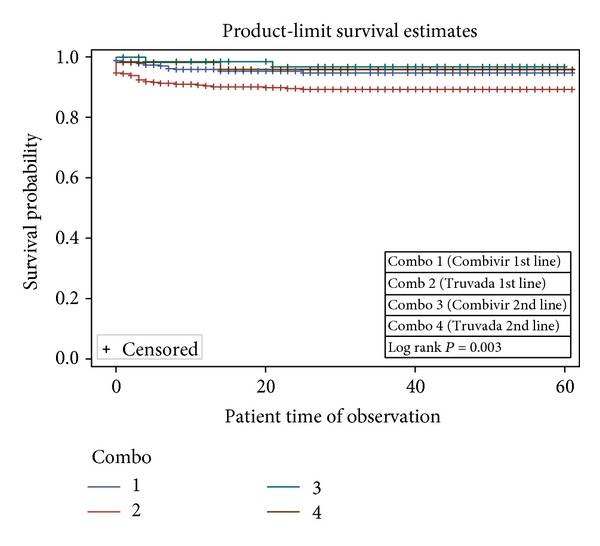
Survival curves based on regimen among patients in NCH.

**Figure 4:**
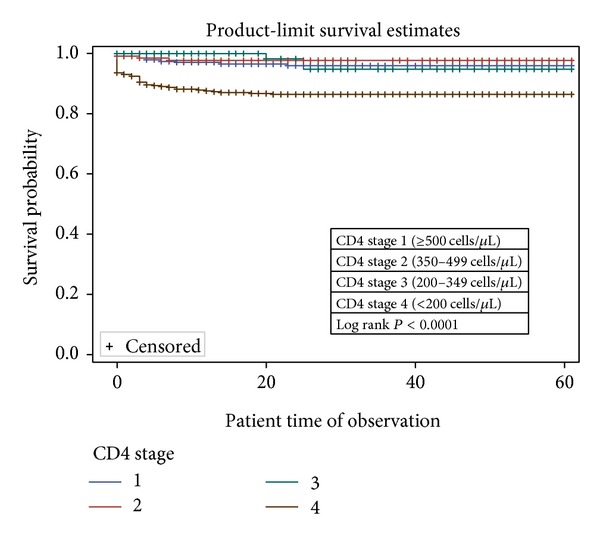
Survival curves based on baseline CD4 staging among patients in NCH.

In the univariable analysis, sex (Figure 1), age group (Figure 2), combination regimen (Figure 3), and baseline CD4 count (Figure 4) were significantly associated with progression to death. Progression to death was 96% higher among patients with baseline CD4 < 200 compared with patients with CD4 > 500 (see Table 2). Patients on Truvada 1st line were more than twice as likely to die compared with those on Combivir 1st line. There was no significant difference in mortality for patients on Combivir- and Truvada-based 2nd line therapy (HR 1.36 95% CI 0.19–9.66). Multivariate analysis using Cox proportional model showed that significant predictors of mortality were age group, initial CD4 staging, drug regimen, and final CD4 staging. Gender was not a confounder with the other variables, and there were no significant interactions among the variables in the study.  Table 3 summarizes the univariate and multivariate analysis.

## 4. Discussion

This 5-year prospective cohort study of people living with HIV/AIDS sheds light on survival and its determinants in a program administered by a rural Nigerian hospital. The mortality rate of 7.6% in this cohort is higher than that reported by Ladep et al, but much lower than the 31% reported by a study in northern Nigeria [6, 11]. The mortality rate was also lower than that reported by studies in other African countries with similar settings [10]. Although this study did not focus on the cause of death, evidence shows that mortality among PLWHA is largely attributable to Tuberculosis coinfection, wasting syndrome, acute bacterial infections, malignancies, and immune reconstitution inflammatory syndrome (IRIS) [10–12].

The proportion of patients lost to followup in this study (15.3%) is higher than what has been reported in other African studies such as in Cameroon (5%) and Tanzania (9.7%), but it is comparable to the rates reported in studies conducted in southeastern Nigeria [2, 4]. LTFU in this study may be explained by the deplorable condition of the major access road leading to the hospital, although this is better reviewed in another publication. The hazard of mortality also was significantly higher among people with greater disease burden (CD4  <  200), similar to what has been reported in literature [2, 4, 6].

Drug combination using highly active antiretroviral therapy (HAART) has improved the survival of PLWHA  [13, 14]. There is evidence that Truvada (or tenofovir-containing regimens) may be superior to Combivir, in terms of fewer adverse effect, improved hemoglobin, and easier dosing [14]. Mortality was almost twice more likely among patients on Truvada-based 1st line therapy compared with Combivir-based therapy (95% CI 1.05–3.36) (see Table 2). This is different from what Velen et al. [7] reported, although that study compared single-agent tenofovir, zidovudine, and stavudine. Bendavid et al. [5] also suggested that Truvada first line had better survival and tolerance profile when compared with Combivir; however that study was based on mathematical simulations [5]. Stratified analysis revealed that patients with CD4 < 200 had the higher hazard of mortality among those on Truvada 1st line. There was no significant difference in mortality for patients on Combivir- and Truvada-based 2nd line therapy (HR 1.36 95% CI 0.19–9.66). This is in line with the findings from a Cochrane review in 2010 [14].

There are limitations to this study. The inability of the study to account for switching therapy from first line to second line regimens is considered a limitation. It would be expected that knowing the time interval from treatment initiation to switching therapy would give valuable clue to outcome based on first line agents only. Also, the high rate of censoring (being live and loss to follow-up) might have biased the study findings. Many of the cases lost to followup had poorer prognosis (low CD4 count); considering them as censored may have resulted in selection bias which could threaten the validity of our findings. This can be improved through intensive patient tracking and timely data management entry. This study did not consider clinical indicators of patient progress (clinical staging), which may be a more suitable predictor of survival.

## 5. Conclusion

The study has shown the predictors of mortality in a cohort of PLWHA followed for 60 months. The patient demographics were comparable to those published in the literature for Nigeria. Mortality in this treatment program was lower than most reported for Nigeria and other African countries. Male gender, CD4 < 200, Non-ART clients, and age above 45 years were among the significant predictors of morality. Hazard of mortality was significantly higher for patients on Truvada-based first line regimen compared to Combivir 1st line; patients with CD4 < 200 had worst outcome among those on Truvada 1st line. The ART program at Nigerian Christian Hospital has improved the survival of its clients, as it had mortality of less than 8% of the study population although more than 15% were lost to follow-up.

It is recommended that managers of ART programs in similar settings should intensify patient follow-up, especially for males and those with low CD4 counts, in order to improve survival. This hospital would need to work towards reducing the rate of loss to follow-up. Further research will be needed to further test the clinical efficacy of Truvada-based regimen, using other outcomes such as progression of CD4 counts.

## Figures and Tables

**Table 1:** WHO staging of HIV based on CD4 cells count.

HIV-associated immunodeficiency	Age-related CD4 values			
	<11 months (%CD4+)	12–35 months (%CD4+)	36–59 months (%CD4+)	>5 years (cells/*μ*L or %CD4+)
None or not significant	>35	>30	>25	>500
Mild	30–35	25–30	20–25	350–499
Advanced	25–29	20–24	15–19	200–349
Severe	<25	<20	<15	<200 or <15%

**Table 2:** Baseline characteristics and associated mortality among 1069 PLWHA in Nigerian Christian Hospital.

Characteristics	Number of patients	Number of deaths (%)
Last known status		
Dead	78 (7.3%)	
Alive	820 (76.9%)	
Loss to followup (LTFU)	168 (15.6%)	
Age group (years)		
15–24	39	1 (2.5)
25–34	354	15 (4.2)
35–44	442	39 (7.0)
45+	234	39 (13.3)
Baseline CD4 staging (cells/*μ*L)		
>500	312	10 (3.2)
350–499	134	3 (2.2)
200–349	111	3 (2.7)
<200	512	62 (12.1)
Drug regimen		
Combivir-based 1st line	383	15 (3.9)
Truvada-based 1st line	548	55 (10.0)
Combivir-based 2nd line	67	2 (2.0)
Truvada-based 2nd line	53	2 (3.8)

**Table 3:** Hazard ratios of mortality according to predictors for PLWHA in Nigerian Christian Hospital.

Variable	Unadjusted (univariate)		Adjusted (multivariate)	
	HR (95% CI)	*P*	HR (95% CI)	*P*
Sex (female versus male)	0.53 (0.34–0.83)	<0.01		
Age group (versus 15–24 years)				
25–34 years	1.59 (0.21–12.06)	0.65	1.25 (0.16–9.61)	0.83
35–44 years	2.59 (0.35–18.96)		1.89 (0.26–13.89)	0.53
45+ years	4.9 (0.63–4.06)	0.12	3.60 (0.49–26.56)	0.21
Baseline CD4 staging (versus >500 cells)				
350–499	0.65 (0.18–2.36)	0.51	0.66 (0.18–2.40)	0.53
200–349	0.82 (0.23–2.97)	0.33	0.91 (0.25–3.30)	0.88
<200	1.96 (2.03–7.74)	<0.01	3.61 (1.84–7.07)	<0.01
Drug combination				
Truvada versus Combivir 1st line	2.36 (1.33–4.19)	<0.01	1.88 (1.05–3.36)	0.03
Truvada versus Combivir 2nd line	1.33 (1.36–0.19)	0.78	1.36 (0.19–9.66)	0.76

## References

[1] WHO *AIDS Epidemic Update 2009*.

[2] Alemu AW, Sebastián MS (2010). Determinants of survival in adult HIV patients on antiretroviral therapy in Oromiyaa, Ethiopia. *Global Health Action*.

[3] FMOH National Guidelines for HIV and Aids Treatment and Care in Adolescents and Adults. http://www.who.int/hiv/pub/guidelines/nigeria_art.pdf.

[4] Onoka CA, Uzochukwu BS, Onwujekwe OE (2012). Retention and loss to follow-up in antiretroviral treatment programmes in southeast Nigeria. *Pathogens and Global Health*.

[5] Bendavid E, Grant P, Talbot A, Owens DK, Zolopa A (2011). Cost-effectiveness of antiretroviral regimens in the World Health Organization's treatment guidelines: a South African analysis. *AIDS*.

[6] Ogoina D, Obiako RO, Muktar HM (2012). Morbidity and mortality patterns of hospitalised adult HIV/AIDS patients in the era of highly active antiretroviral therapy: a 4-year retrospective review from Zaria, Northern Nigeria. *AIDS Research and Treatment*.

[7] Velen K, Lewis JJ, Charalambous S, Grant AD, Churchyard GJ, Hoffmann CJ (2013). Comparison of tenofovir, zidovudine, or stavudine as part of first-line antiretroviral therapy in a resource-limited-setting: a cohort study. *PLoS ONE*.

[8] WHO Organization (2010). *Antiretroviral Therapy for HIV Infection in Adults and Adolescents: Recommendations for a Public Health Approach-2010 Revision*.

[9] DEFINITIONS WC HIV/AIDS Programme.

[10] Johannessen A, Naman E, Ngowi BJ (2008). Predictors of mortality in HIV-infected patients starting antiretroviral therapy in a rural hospital in Tanzania. *BMC Infectious Diseases*.

[11] Ladep G, Agbaji OO, Agaba PA (2013). Hepatitis B co-infection is associated with poorer survival of HIV-infected patients on highly active antiretroviral therapy in West Africa. *Journal of AIDS & Clinical Research*.

[12] Lawn SD, Myer L, Orrell C, Bekker L, Wood R (2005). Early mortality among adults accessing a community-based antiretroviral service in South Africa: implications for programme design. *AIDS*.

[13] Temesgen Z, Warnke D, Kasten MJ (2006). Current status of antiretroviral therapy. *Expert Opinion on Pharmacotherapy*.

[14] Humphreys EH, Chang LW, Harris J (2010). Antiretroviral regimens for patients with HIV who fail first-line antiretroviral therapy. *Cochrane Database of Systematic Reviews*.

